# The Impact of Freeze-Drying Conditions on the Physico-Chemical Properties and Bioactive Compounds of a Freeze-Dried Orange Puree

**DOI:** 10.3390/foods9010032

**Published:** 2019-12-30

**Authors:** Marilú A. Silva-Espinoza, Charfedinne Ayed, Timothy Foster, María del Mar Camacho, Nuria Martínez-Navarrete

**Affiliations:** 1 Food Technology Department, Food Investigation and Innovation Group, Universitat Politècnica de València, Camino de Vera s/n, 46022 Valencia, Spain; masiles@doctor.upv.es (M.A.S.-E.); mdmcamvi@tal.upv.es (M.d.M.C.); 2 Department of Food, Nutrition and Dietetics, School of Biosciences, University of Nottingham, Sutton Bonington Campus, Loughborough LE12 5RD, UK; Charfedinne.Ayed@nottingham.ac.uk (C.A.); Tim.Foster@nottingham.ac.uk (T.F.)

**Keywords:** vitamin C, total phenols, total carotenoids, antioxidant activity, colour, mechanical properties, pressure, shelf temperature, freezing rate

## Abstract

Fruits are essential for a healthy diet, as they contribute to the prevention of cardiovascular diseases and some cancers, which is attributed to their high bioactive compound content contributing to their antioxidant capacity. Nevertheless, fruits have a short shelf life due to their high-water content, and freeze-drying is a well-known technique to preserve their nutritive quality. However, it is an expensive technology, both due to the use of low pressure and long processing time. Therefore, an optimisation of variables such as the freezing rate, working pressure and shelf temperature during freeze-drying may preserve fruit quality while reducing the time and costs. The impact of these variables on colour, porosity, mechanical properties, water content, vitamin C, total phenols, β-carotene, and antioxidant activity of a freeze-dried orange puree was evaluated. The results showed a great impact of pressure and shelf temperature on luminosity, chroma and water content. Vitamin C and β-carotene were more preserved with higher shelf temperatures (shorter times of processing) and lower pressure, respectively. The optimum freeze-drying conditions preserving the nutrients, and with an interesting structural property, perceived as a crunchy product by consumers, are low pressure (5 Pa) and high shelf temperature (50 °C).

## 1. Introduction

It is known that there is interest in the consumption of fruits, as they are recommended as components of a healthy diet due to their contribution to the prevention of some diseases when they are consumed in adequate quantity [1,2]. This effect is attributed to their high content of bioactive compounds such as phytochemicals, some vitamins and fibre [3]. In particular, the orange and its derived products are a rich source of flavonoids (mainly hesperidin), carotenes, and vitamin C, with concentrations in the range of 15–238.8 mg, 182–198 µg and 43.5–50 mg/100 g edible fruit, respectively [4,5,6,7,8,9]. In fact, an average orange would contribute 80% of the RDA (recommended daily allowance) of vitamin C [10]. However, fruits have two main problems that affect their continuous availability, which are seasonality and short shelf life. Dehydration is one of the most common techniques used to preserve food. In addition, it also entails a reduction in the volume and weight of the product, which facilitates its transport and handling [11].

Freeze-drying is a dehydration technique based on the sublimation of the water present in a product, which results in a reduction of water activity and therefore the related deterioration processes to which a food is subjected [12]. The product is frozen in order to be subjected to vacuum pressure with the consequent sublimation and desorption of the water. Freeze-drying operates at low temperatures, which contributes to preserve characteristics such as taste, colour or appearance and to minimize the degradation of thermolabile compounds, many of them responsible for the aromas and nutritional value of the fruits. Thus, the final freeze-dried product is high quality as compared with other techniques of dehydration [13].

Despite the improved microbiological stability of the final product, the chemical and physical attributes may be sometimes compromised. On the one hand, the high porosity and the low water content of the freeze-dried products make the interaction between the solutes and the oxygen at the end of the process more accessible. In this way, the oxidation of bioactive compounds, such as vitamin C, phenols or carotenoids may be promoted. On the other hand, the physical problems are related to the glass transition of the amorphous matrix, which is usually developed during the freeze-drying process. Above the glass transition temperature (Tg), the change from the more stable glassy state to the rubbery state occurs [14]. Freeze-dried fruit pulps, as sugar-rich foods, have a low Tg value in the range of 5–15 °C [15,16]. For this reason, they present collapse and other structural problems related to stickiness and caking, which begin to be developed about 20 °C above Tg [14]. A usual way to delay these problems is the incorporation of high molecular weight biopolymers that contribute to an increase in Tg, or that exert a steric role [17,18].

The disadvantage of freeze-drying is its high cost, due to the long process times and the energy cost related to the vacuum stage. For this reason, it has only been widely used to obtain products with high value added, as occurs in the pharmaceutical industries as well as in some specific food industries, such as rehydratable coffee. However, given the high sensory and functional value of fruits, associated with their high content of bioactive compounds, freeze-drying can be a niche opportunity in this case. In this sense, the technique can provide different food formats, among them, a crunchy fruit product with good consumer acceptance as a snack [18]. Despite adequate optimization of the process conditions contributing to reduce the duration of the process, several reports have indicated that both the freezing and the drying variables, such as the freezing rate or the working pressure and shelf temperature during the drying step, may affect the quality of the obtained product [18,19,20,21,22,23,24]. As regards the impact of increasing the shelf temperature, a study carried out with grapefruit puree indicated a decrease of more than 50% in drying time when increasing the temperature up to 40 °C, without a great impact on aspects such as colour, texture or vitamin C content [25]. Nor was an effect observed on the vitamin C content when a mandarin juice was freeze-dried at 40 °C compared to that processed at room temperature [18]. Nevertheless, the shelf temperature should not exceed either the collapse temperature or that which could cause damage to the thermolabile compounds of interest.

In this study, the impact of freeze-drying conditions on the quality of a freeze-dried orange puree with added gum Arabic and bamboo fibre was evaluated. Two freezing rates (conventional and blast freezer), three different shelf temperatures (30, 40, 50 °C) and two working pressures (5 and 100 Pa) were combined. The quality indices measured were the water content, colour, porosity, mechanical properties, vitamin C, carotenoids and phenolic content, as well as the total antioxidant capacity.

## 2. Materials and Methods

### 2.1. Raw Materials

Oranges (*Citrus x sinensis* cultivar Navel) used in this study were selected by subjective visual inspection based on a similar weight and size colour homogeneity and good physical integrity (absence of external physical damage). They were bought in October 2019 from a local supermarket in the city of Valencia (Spain) and immediately processed. Carriers used to obtain the dehydrated orange samples were gum Arabic (GA, Scharlab, Sentmenat, Spain) and bamboo fibre (BF, VITACEL^®^, Rosenberg, Germany).

### 2.2. Freeze-Drying Processing

Oranges were washed, peeled, cut and triturated in a bench top electrical food processor for 40 s at speed 4 (2000 rpm) followed by 40 s at speed 9 (9100 rpm) (Thermomix TM 21, Vorwerk, Spain). The orange puree was mixed for 10 min at speed 3 (1000 rpm) with (5 g GA + 1 g BF)/100 g orange puree as to ensure the physical stability of the dried product [26]. The formulated orange puree (FOP) was distributed in 10.5 × 7.8 cm aluminum plates of 0.5 cm thickness. Samples were immediately frozen at two different freezing rates (FR): slow freezing (FR-S) in a conventional freezer (Liebherr Mediline LGT 2325, Liebherr, Baden-Wurtemberg, Germany) for 48 h and fast freezing (FR-F), where the samples were frozen for 3 h at −38 °C in a blast freezer (Hiber RDM051S, Hiber, Cernusco sul Naviglio, Italy), and then stored at −45 °C in the conventional freezer for at least 24 h. Frozen samples were dried in a freeze-drier (Telstar Lyoalfa-6, Telstar, Terrassa, Spain) at different pressures (P) in the chamber and shelf temperatures (T). Twelve different conditions were studied (Table 1). The shelf temperature conditioned the drying time, this being 25 h at 30 °C, 7 h at 40 °C and 6 h at 50 °C. The time was selected based on preliminary experiments to be enough to achieve a water content lower than 4%. At these conditions, the physical stability of the formulated puree was known to be guaranteed, as no structural collapse was observed.

### 2.3. Water Content

The water content (x_w,_ g water/100 g product) of FOP was determined using the AOAC method [27]. The sample was dried in a vacuum oven (Selecta^®^, Vaciotem-T, J.P. Selecta S.A., Barcelona, Spain) at 60 ± 1 °C under *P* < 100 mm Hg until constant weight (XS204 DeltaRange^®^, Mettler Toledo, Switzerland). For the freeze-dried puree, an automatic Karl Fisher titrator (Mettler Toledo, Compact Coulometric Titrator C10S, Worthington, OH, USA) was used to obtain the water content. Triplicates were performed in each case.

### 2.4. Mechanical Properties

The mechanical behaviour of the freeze-dried puree was registered using a texture analyser (TA-XT2i, Stable Micro Systems, Godalming, UK). Portions of 20 × 20 mm of the freeze-dried puree were compressed using a cylindrical probe of 10 mm diameter, applying a strain of 80% with a test speed of 1 mms^−1^. Six replicates were performed per sample. The parameters analysed in the test were the force required to fracture the sample (Fracture force, F_f_), expressed in Newtons, and the slope (S, N/mm) of the curve in the linear zone prior to fracture point, related to the sample resistance to deformation (rigidity) [28].

### 2.5. Colour Measurements

The CIE L*a*b colorimetric space was considered to characterize the colour [29]. A colorimeter (Minolta, CM 3600D, Japan) was used to measure the colour of the surface of the freeze-dried puree, taking the system observer 10° and illuminant D65 as reference. Colour coordinates, L*, a*, b*, were obtained for each freeze-dried puree. From them, the hue angle (h*, Equation (1)) and chroma or saturation (C*, Equation (2)) were obtained. When total colour differences (∆E*) were calculated, Equation (3) was used. Measurements were carried out with the specular component excluded. Six replicates were performed per sample.

h* = arctan(b*/a*) (1)

C* = (a*^2^ + b*^2^)^0.5^ (2)

∆E* = ((∆L*)^2^ + (∆a*)^2^ + (∆b*)^2^)^1/2^ (3)

### 2.6. Porosity

True density (ρ) and apparent density (ρ_a_) were obtained in order to obtain the porosity (ε, %). True density was calculated based on the sample composition (Equation (4)). Portions of the cakes were obtained with a punch of 22 mm diameter, and were exactly measured in height and diameter with a calliper. Apparent density of each portion was calculated from the weight (m, g; XS204 DeltaRange^®^, Mettler Toledo, Switzerland) and corresponding volume (V, cm_3_) (Equation (5)). The porosity was calculated from Equation (6).

(4) $$\rho \;=\;\frac{1}{\frac{X_{w}}{\rho_{w}}\;+\;\frac{X_{\mathrm{CH}}}{\rho_{\mathrm{CH}}}}$$

where x_w_ and x_CH_ are the mass fractions of the two main components of each sample (water and carbohydrates, respectively, x_w_ was determined as described in Section 2.3, and x_CH_ by difference); ρ_w_ and ρ_CH_ are their densities (ρ_CH_ = 1.4246 g/cm^3^, ρ_w_ = 0.9976 g/cm^3^ [30]).

(5) $$\rho_{a\;}=\;\frac{m}{v}$$

(6) $$\varepsilon \left(\%\right)=\;100\frac{\rho -\rho_{a}}{\rho }$$

### 2.7. Total Polyphenolic Compounds

The extraction of total phenolic compunds (TP) was carried out according to Tomás-Barberán et al. [31] with minor modifications. FOP (2.5 g) or freeze-dried puree (0.5 g) were mixed with 9 mL of methanol:water (70:30) using a magnetic multi-stirrer at 200 rpm (JEIO TECH Lab Companion MS-51M, JEIO TECH Lab Companion, Seoul, Korea) under darkness and at room temperature for 30 min. The homogenates were centrifuged at 11,515× *g* at 4 °C for 10 min (GYROZEN Co., 1236R, GYROZEN, Daejeon, Korea). The supernatant was collected and analysed as to TP using the Folin–Ciocalteu method, which was adapted from Benzie et al. [32] with some modifications as described by Selvendran et al. [33]. The TP content was calculated as mg of gallic acid equivalents (GAE)/100 g dry basis (db) sample, using a standard curve in the range of 0–1000 ppm of gallic acid (Sigma-Aldrich, Saint Louis, MO, USA). In this study, all bioactive compounds were referred to the percentage (%) of the corresponding bioactive compound preserved in the freeze-dried puree (FDP) in reference to the FOP, calculated based on Equation (7). This test was done in triplicate for each sample.

(7) $$\mathrm{PBc}\;\left(\%\right)\;=\;\frac{{\mathrm{Bc}}_{\mathrm{FDP}}}{{\mathrm{Bc}}_{\mathrm{FOP}}}\times 100$$

where PBc (%) is the percentage of the corresponding bioactive compound preserved; Bc_FDP_ is the bioactive compound content in the freeze-dried puree (mg/100 db); and Bc_FOP_ is the bioactive compound content in the formulated orange puree (mg/100 g db).

### 2.8. Antioxidant Activity

The antioxidant activity (AOA) was determined with the DPPH and FRAP tests. The methanolic extract obtained for the quantification of TP was used to this end. DPPH was carried out according to Brand-Williams et al. [34] with minor modification. For these samples, the steady state of the reaction was reached at 15 min, when the absorbance at 515 nm was measured again. The FRAP test was carried out according to Benzie et al. [32]. The results for both methods were converted to mmol Trolox equivalents/100 g db freeze-dried puree. The AOA was also expressed as the percentage (%) of this activity preserved in the FDP in reference to the FOP (Equation (7)). Three replicates were performed per sample.

### 2.9. Vitamin C

Total vitamin C content (VC) was determined by the reduction of dehydroascorbic acid to ascorbic acid (AA) using high-performance liquid chromatography (HPLC) (Jasco, Italy). The reduction was carried out by mixing 0.5 g of FOP or 0.075 g of each of the 12 freeze-dried puree samples with 2 mL of a 20 g/L DL-dithiothreitol solution (Scharlab, Spain) for 2 h at room temperature and under darkness [35,36]. The extraction of the mixture was carried out according to Xu et al. [37]. The HPLC conditions were: Kromaphase100-C18, 5 mm (4.6 × 250 mm) column (Scharlab SL); mobile phase 0.1% oxalic acid, volume injected 10 μL, flow rate 1 mL/min, detection at 243 nm (detector UV-visible MD-1510, Jasco, Cremella, Italy) at 25 °C. A standard solution of L (+) ascorbic acid (Scharlab SL, Sentmenat, Spain) in the range of 5–200 ppm was prepared. The VC content was calculated as mg AA/100g db sample and the percentage (%) of this bioactive compound preserved in the FDP in reference to the FOP was calculated (Equation (7)). Three replicates were performed per sample.

### 2.10. β-Carotene

The extraction of β-carotene (BC) was performed using the method of Olives et al. [38] with some modifications. FOP (0.8 g) or freeze-dried puree (0.2 g) were mixed with 9 mL of hexane/ethanol/acetone (50:25:25, *v*/*v*/*v*) using a magnetic multi-stirrer at 200 rpm (JEIO TECH Lab Companion MS-51M, Korea), under darkness and at room temperature for 30 min. The homogenates were centrifuged at 11,515× *g* at 4 °C for 10 min (GYROZEN Co., 1236R, Daejeon, Korea). Distilled water was added to the supernatant (10 mL distilled water/100 mL supernatant) and was manually stirred for 2 min. The absorbance of the upper layer was measured at 446 nm (spectrophotometer V-1200 VWR, VWR, Radnor, PA, USA) [38]. The BC was calculated as mg BC/100 g db sample using a β-carotene (Dr. Ehrenstorfer, Augsburg, Germany) calibration curve in the range of 0.5–7 ppm. The BC was referred to the percentage (%) of this bioactive compound preserved in the FDP in reference to the FOP (Equation (7)). Three replicates were performed per sample.

### 2.11. Statistical Analysis

Data were subjected to Partial Least Squares Regression (PLS-R) and a three way analysis of variance (ANOVA) using Tukey’s HSD test to establish the significant effect of shelf temperature, pressure and freezing rate on the parameters studied, with 95% confidence interval, by using XLSTAT statistical and data analysis solution (Addinsoft, 2019, Long Island, NY, USA). F-Values obtained with the ANOVA were also considered to identify the most important factors. Furthermore, a Pearson’s correlation analysis between antioxidant capacity and the bioactive compounds was carried out.

## 3. Results and Discussion

All the results obtained are detailed in the Supplementary Materials (Tables S1–S3). The most relevant aspects are detailed below.

### 3.1. Physicochemical Characterization

**Figure 1:**
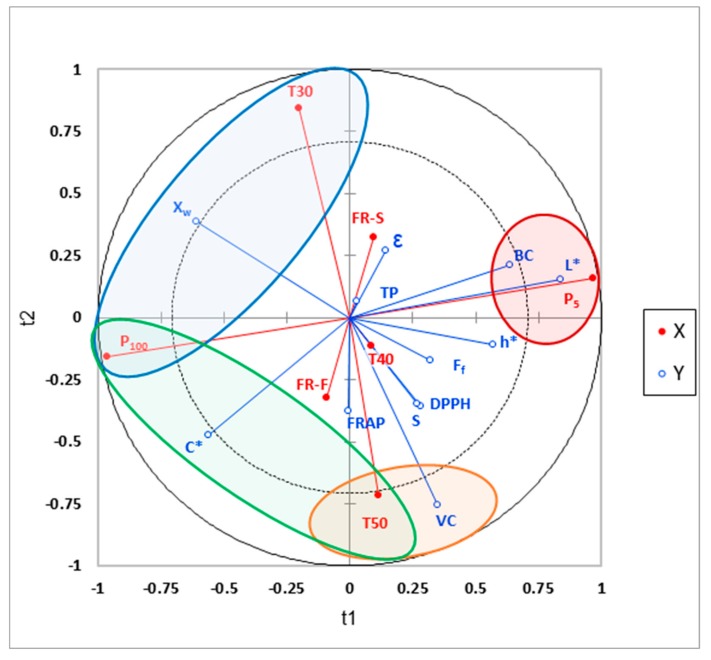
PLS-R projections of freeze-dried orange puree at a range or pressure, temperature and rate of freezing conditions. The dependent variables (X) are projected in red and the qualitative explanatory variables (Y) are projected in blue. The variables Y significantly correlated with a specific dependent variable are circled in red, blue, orange and green. Variables X: T30, T40, T50: shelf temperature applied during freeze-drying at 30, 40 and 50 °C, respectively; P_5_ and P_100_: working pressures of 5 Pa and 100 Pa respectively; FR-S: slow freezing rate and FR-F: fast freezing rate. Variables Y: percentage (Equation (7)) of TP: total phenols, VC: vitamin C, BC: beta carotene, antioxidant activity (FRAP and DPPH); F_f_: fracture force (N); S: slope related to rigidity (N/mm); Xw: water content (g water/100 g sample); **ε**: porosity (%).

The experimental results of the colour characterization, mechanical properties, porosity, and water content of the freeze-dried purees obtained under each of the 12 studied conditions were processed by PLS-R (Figure 1 and Table S4). Axis 1 (t1) mainly represents the impact of pressure on the qualitative explanatory variables (Y), while axis 2 (t2) represents the impact of shelf temperature on Y. The vectors of freezing rate are in the inner circle, which indicates that in general, these factors are not significantly correlated with the different studied properties of the samples. The variables Y that are significantly affected by a specific dependent variable (X) are circled in red, blue, orange and green.

#### 3.1.1. Colour

The PLS–R revealed a good correlation between pressure and L*, C*, h* values. They can be observed according to axis 1: P_5_ projected on the positive side while P_100_ is projected on the negative side. The external and near position of P_5_ to L* and P_100_ to C* denotes a significant effect of pressure on colour attributes, so that low pressure leads to a high L* value (see red circle in Figure 1) while high pressure leads to a high C* value. Despite h* also being positively projected on axis 1 and so affected by pressure, its projection stays in the inner circle of the PLS-R although near the external limit. This indicates a lowered impact of the pressure on the hue angle when compared with that observed for C* and L*. It can also be notice that the projection of C*on the PLS-R, in the negative part of axis 1 and axis 2, is higher when the interaction between P_100_ and T50 is considered (see green circle in Figure 1). The shorter freeze-drying process carried out at 50 °C contributes to promoting freeze-dried products with a higher value of chroma. In addition, C* is projected in the same side of the fast freezing rate (FR-F), the last one being in the inner circle (poorly correlated according to axis 2).

**Figure 2:**
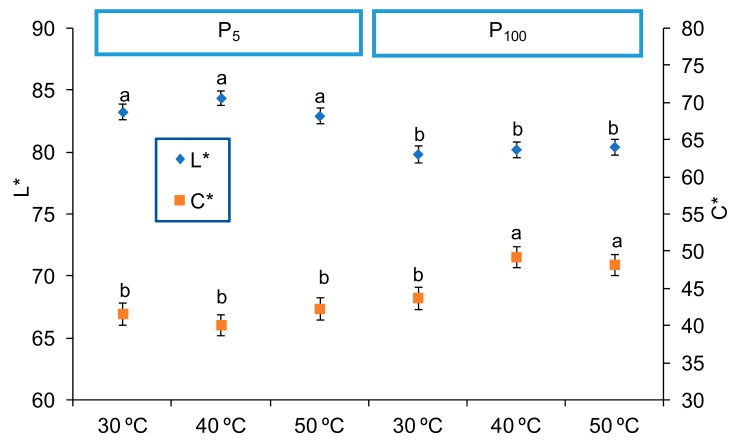
Values (mean and Tukey’s HSD) of L* in left axis and C* in right axis of the freeze-dried purees according to the interaction between shelf temperature (30, 40 or 50 °C) and pressure (P_5_: 5 Pa and P_100_: 100 Pa) factors. Different letters for each attribute indicate different homogeneous groups for the Temperature*Pressure interaction (*p* < 0.05). Data of both freezing rates are considered in the mean values.

These observations were confirmed by the ANOVA, as values of L*, C* and h* of the samples were significantly affected by working pressure (*p* < 0.05). Further, C* was also affected by the interaction between shelf temperature and freezing rate (*p* < 0.05), but with a low F-Value (7.86). Taking into account the significances shown by the PLS-R analysis and the F-Values of the ANOVA, Figure 2 was constructed, showing L* and C* values of the samples obtained at the different pressure and shelf temperature and considering the mean value at both freezing rates. When working with the highest pressure during freeze-drying (P_100_), the samples showed lower values of L* and higher C*, which means a darker and saturated colour. In this case, the chroma is specially enhanced at a higher temperature in the freeze-drier shelves, either 40 °C or 50 °C (Figure 2, *p* < 0.05). The hue angle, with values between 80.3 and 82.6, showed a lower value when working with high pressure and freeze-drier shelves temperature below 50 °C (*p* < 0.05). Similar results have been reported by Hammami et al. [22], who noted a slight L* decrease when working at higher pressures (*P* > 108 Pa) for strawberries pieces, which was related to the pronounced shrinkage observed under these conditions. Different authors found that the operating working pressure should be lower than 50 Pa to avoid shrinkage for strawberries pieces and banana slides [22]. With regards to shelf temperature, increasing temperature may cause some slight sugar browning reactions like non-enzymatic or Maillard reactions, which means a reinforcement of the colour shown by the increase in C*. Nevertheless, it is better detected in shrunken samples due to its different optical light reflection capacity [22].

According to the PLS-R and the ANOVA, pressure and shelf temperature were the factors that had a significant impact on the colour. The total differences in colour were calculated in order to evaluate the impact of both factors on the colour. Regarding the shelf temperature, values of ΔE* between 1.11 and 5.51 were obtained, while the range was 4.45–9.98 for the pressure influence. According to Bodart et al. [39], total differences in colour are not obvious to the human eyes when ΔE* < 1, minor colour differences could be appreciated by the human eye depending on the hue when 1 < ΔE* < 3 and visually obvious changes for human eyes occur when ΔE* > 3. Therefore, it seems that pressure has a greater effect than the other factors on the colour.

#### 3.1.2. Structure

Freeze-dried purees showed high porosity, typical of freeze-dried products, with values ranging from 86.4–87.4%. The PLS–R indicated a correlation between FR-S and porosity (mainly projected in the positive side of axis 2, Figure 1), which means that a slow rate of freezing leads to bigger ice crystals and so a bigger expansion of gas cells in the structure (higher porosity) during the freeze-drying process. Nevertheless, they are in the inner circle, which means the effect is not significant. In fact, according to ANOVA, porosity of the samples was not significantly affected by any factor evaluated (*p* > 0.05).

**Figure 3:**
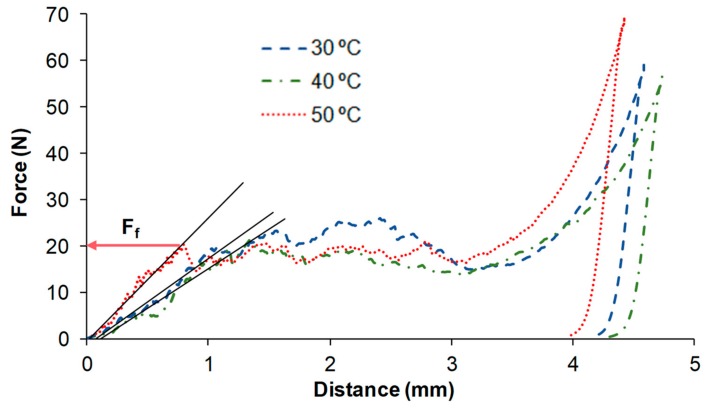
Examples of force–distance curves obtained from the freeze-dried purees frozen at a slow rate and freeze-dried at 5 Pa, and with different shelf temperatures (30, 40 and 50 °C). F_f_: fracture force.

As a result of the mechanical analysis, the force versus distance curves were obtained for each sample freeze-dried under each of the 12 conditions (Figure 3). The linear regression of the first part of the curve was taken to calculate the slope before F_f_, related to the rigidity of the sample. As can be observed in the PLS-R (Figure 1), factors T50 and rigidity are both partially correlated and projected on the negative side of axis 2, which means that a higher temperature leads to an increase in the rigidity of the freeze-dried samples. However, because the rigidity is projected in the inner circle, it indicates a moderate positive correlation. In regards to the ANOVA results, the shelf temperature had a significant effect on the rigidity (slope) of the freeze-dried samples (*p* < 0.05); however, its low F-Value (8.65) makes it possible to confirm its low significance.

Values of the slope 18–26 N/mm were obtained when heating the freeze-drier shelves to 50 °C, as compared to 11–18 N/mm values obtained at 30 and 40 °C. As a greater slope indicates less deformation of the sample by exerting an effort on it, it can be concluded that freeze-drying heating the shelves to 50 °C promotes the mechanical rigidity of the freeze-dried samples before they fracture. This can be considered desirable as it would be related to a higher mechanical resistance of the sample during its handling before consumption.

Fracture force of all the samples varied between 12.1 ± 1.6 N and 19 ± 5 N. The PLS-R revealed a moderate positive correlation between fracture force and both lower pressure (P_5_) (both projected in the positive side of axis 1) and T50 (both projected in the negative side of axis 2). This means that working with lower pressure and higher temperature seems to promote a freeze-dried puree that is more resistant to fracture. According to the ANOVA, neither the temperature nor the freezing rate showed a significant effect on the fracture force (*p* > 0.05). The pressure did show *p* < 0.05, but again, with a very low *F*-Value (7.17).

The water content of the samples ranged between 2.2 ± 0.3 and 4.2 ± 0.2 g water/100 g sample. The PLS-R indicated a high correlation between water content (%), high pressure (P_100_) and low temperature (T30), circled in blue colour in Figure 1. In fact, this statement was confirmed by the ANOVA that indicated a significant effect on the final water content of the freeze-dried samples by the shelf temperature and the pressure (*p* < 0.05). In this way, drying at the highest pressure (100 Pa) and the lowest shelf temperature (30 °C) promoted samples with the highest water content. On the other hand, Figure 1 shows a clear negative correlation between water content and fracture force, which means the smaller the water content of the freeze-dried samples, the greater the mechanical resistance to fracture. In this case, the final water content of the samples was lower than 4.2%. However, it seems that small changes outside this range, related to working conditions, will have an important impact on the mechanical properties of the sample.

### 3.2. Bioactive Compounds

The quantification of total phenols, vitamin C, β-carotene, and antioxidant activity evaluated by two methods (FRAP and DPPH), was carried out in the FOP and after being freeze-dried for each of the 12 conditions evaluated.

**Figure 4:**
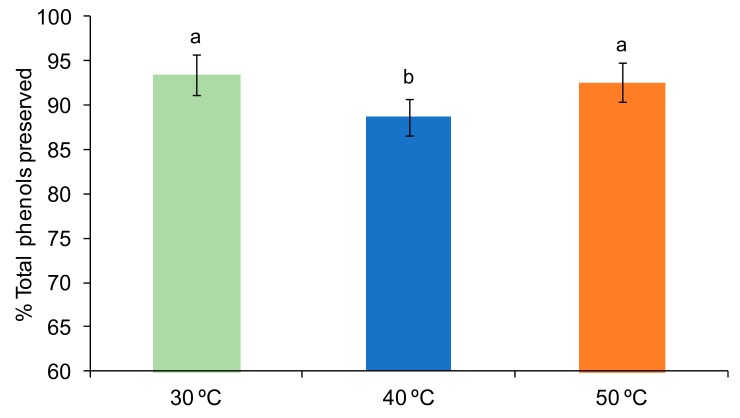
Percentage (%) of preserved total phenols (mean and Tukey’s HSD) of samples according to the shelf temperature factor (30, 40 or 50 °C). Different letters indicate different homogeneous groups for the shelf temperature factor (*p* < 0.05). Data of both freezing rates and both pressures are considered in the mean values.

The PLS-R indicated that TP is not projected on the bi-plot (Figure 1), which means that it is relatively less impacted than the other bioactive compounds (TP vector very close to the origin 0.0). In fact, according to the ANOVA, the TP was not affected by working pressure and freezing rate (*p* > 0.05) and was better preserved when the freeze-drying was carried out at 30 or 50 °C as compared to 40 °C (*p* < 0.05), despite the temperature factor also having a low F-Value (8.07). In fact, the most marked difference was observed with the factor temperature, which was 5% of preservation between 30 and 40 °C (Figure 4). No significant interactions between the factors were observed (*p* > 0.05).

Figure 4 shows TP preservation by shelf temperature, considering both freezing rates and pressures in the mean values. It seems that TP may be affected by the ratio of time and temperature of processing, as reported by other authors [40]. In this case, mild 40 °C heating for more than 7 h seems to compromise TP preservation.

**Figure 5:**
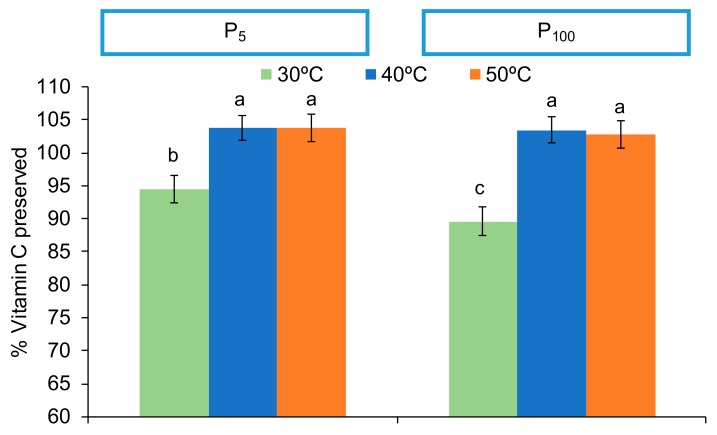
Percentage (%) of preserved vitamin C (mean and Tukey’s HSD) according to the interaction between shelf temperature (30, 40 or 50 °C) and pressure (P_5_: 5 Pa and P_100_: 100 Pa) factors. Different letters indicate different homogeneous groups for the temperature*pressure interaction (*p* < 0.05). Data of both freezing rates are considered in the mean values.

The presence of VC in the final product is used as a reference of high nutritional quality for the different industrial processes, due to its relative instability to heat, oxygen and light [41,42]. The impact of temperature on VC can be clearly observed on the PLS-R according to axis 2 (Figure 1). Vector T50 and T40 are projected in the same direction (negative side of axis 2), while T30 is anti-correlated to them (projected on the positive side of axis 2). This confirms that a higher shelf temperature along the process preserved the vitamin C of the samples. According to the ANOVA, it can be confirmed that VC was affected by the shelf temperature and the pressure during freeze-drying (Figure 5, *p* < 0.05). A significant interaction between both factors indicated that heating the freeze-drier shelves to 40 or 50 °C promoted samples with higher vitamin C content than those freeze-dried at 30 °C. Despite VC being reported to have thermal stability [41], the length of time required when the freeze-drying is carried out at 30 °C (25 h) may cause VC loss. However, the lower content of VC of samples freeze-dried at 30 °C was even lower when higher pressure was applied (Figure 5). This means that for a long expected process time, oxygen presence should be maximally avoided.

**Figure 6:**
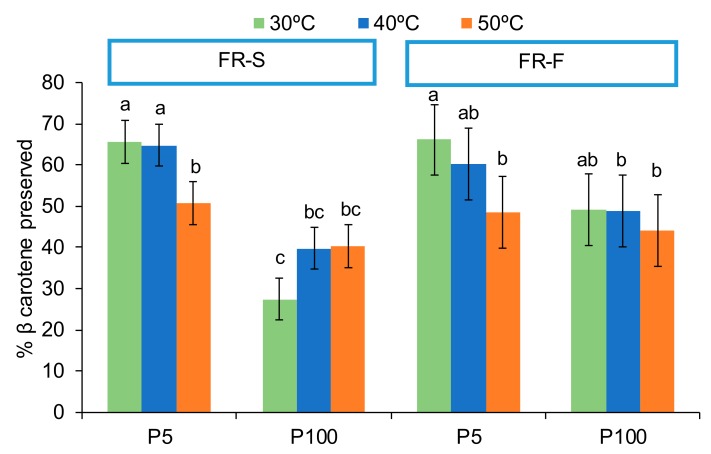
Percentage (%) of preserved β-carotene (mean and Tukey’s HSD) according to the interaction between shelf temperature (30, 40 or 50 °C) and pressure (P_5_: 5 Pa and P_100_: 100 Pa) factors for each freezing-rate (FR-S and FR-F: slow and fast freezing rates, respectively). Different letters indicate different homogeneous groups for the temperature*pressure interaction for both freezing-rate independently (*p* < 0.05).

Certain carotenoids are highly coloured compounds that also exhibit provitamin A activity. BC has the highest vitamin A activity [43]. The PLS-R (Figure 1) underlined, in particular, the BC preservation by low pressure, as it is projected on the positive side of axis 1 and highly correlated with vector P_5_. In this case, the ANOVA indicated a significant effect of the three factors considered (*p* < 0.05, Figure 6), the higher the pressure, the higher the temperature, and the slower the freezing rate, the greater the loss of BC. Nevertheless, the F-Values were 88, 6 and 6 for pressure, shelf temperature and freezing rate, respectively. Again, a low F-Value in the ANOVA is correlated with no significant effect detected by the PLS-R analysis. However, the ANOVA also revealed a significant interaction between the pressure and both the shelf temperature and the freezing rate (*p* < 0.05). Figure 6 shows the interaction of pressure and shelf temperature for each FR.

According to these interactions, the pressure effect is no longer significant at 50 °C, when a significant part of BC has already been degraded by the effect of the shelf temperature. In addition, the effect of the shelf temperature was not significant at higher pressure. Furthermore, when almost no oxygen is present (P_5_), BC is conserved quite well, regardless of freezing rate. It is in the greater presence of oxygen (P_100_) when the effect of the freezing rate is significant in relation to the better preservation of BC at the FR-F. From the ANOVA results, the PLS-R analysis can be nuanced in the sense that the most recommendable way to keep the maximum carotenoids present in the orange puree during freeze-drying is when the drying stage is carried out at the lowest pressure studied and heating the freeze-dryer shelves to 30 or 40 °C, without the freezing-rate being relevant in this case.

As regards antioxidant activity, values of DPPH between 86.5 ± 1.8% and 94.3 ± 1.5% were observed. The PLS-R revealed that the vector DPPH is projected on the lower right corner of the graph, which means a moderate positive correlation with both the lowest pressure and the highest temperature. According to the ANOVA analysis, no significant effect of pressure was detected (*p* > 0.05). Although freeze-drying carried out at 30 °C leads to samples with lower DPPH than those processed at 40 °C or 50 °C (p < 0.05), once again, with a low F-Value (9.51) for shelf temperature factor. On the other hand, FRAP values between 91 ± 2% and 103 ± 6% of preservation for all the conditions studied were analysed. Neither the PLS-R nor the ANOVA analysis showed a significant effect of any of the freeze-drying process variables on FRAP (*p* > 0.05).

From the Pearson correlation, only a significant and positive correlation (0.5774, *p* < 0.05) was obtained between values of DPPH and vitamin C. Despite AOA being correlated in a positive way with the total phenolic, vitamin C content and carotenoids, it has been suggested that VC contributes to antioxidant capacity more than others antioxidant constituents, such as phenols or carotenoid in fruits with high VC content [41,44]. This can also be observed on the PLS-R projection as VC, DPPH and FRAP are projected on the same direction.

## 4. Conclusions

In conclusion, the optimum freeze-drying conditions for preserving the nutrients considered in this study and with interesting structural properties of the obtained product, as to be perceived as crunchy by the consumers, are low pressure (5 Pa) and high shelf temperature (50 °C). These conditions also promote freeze-dried puree with a clear, yellowish and less saturated colour. The fact that a lower degradation of nutrients was observed at higher temperatures may be explained by the great reduction (75%) of the duration of freeze-drying process at 50 °C, and the mild temperatures used. The shorter exposure of nutrients to a minimal presence of oxygen in a high porous matrix is less favourable to oxidation/degradation reactions and contributes to the preservation of nutrients. As regards to the statistical analysis of the data obtained in this study, PLS-R projection may be recommended against ANOVA as an easier tool to detect the most important factors and interactions to be considered for freeze-drying process optimization. ANOVA allows a more precise analysis, though less practical.

## Figures and Tables

**Table 1:** Sample and conditions code according the 12 different freeze-drying conditions studied.

Sample Code	Shelf Temperature (T)			Pressure (P)		Freezing Rate (FR)	
	30 °C	40 °C	50 °C	5 Pa (P_5_)	100 Pa (P_100_)	Slow (S)	Fast (F)
S_30_P_5_	X			X		X	
F_30_P_5_	X			X			X
S_30_P_100_	X				X	X	
F_30_P_100_	X				X		X
S_40_P_5_		X		X		X	
F_40_P_5_		X		X			X
S_40_P_100_		X			X	X	
F_40_P_100_		X			X		X
S_50_P_5_			X	X		X	
F_50_P_5_			X	X			X
S_50_P_100_			X		X	X	
F_50_P_100_			X		X		X

## Supplementary Materials

The following are available online at https://www.mdpi.com/2304-8158/9/1/32/s1, Table S1: Values (mean ± SD) of the different physicochemical properties evaluated. All acronyms used are described in the main text. Table S2: Percentage (mean ± SD) of the bioactive compounds preserved in the FDP for each condition evaluated. All acronyms used are described in the main text. Table S3: Values (mean and Tukey’HSD classification) of the different physicochemical properties and bioactive compounds evaluated for: each individual factor (a–c), the interaction between two different factors (d–f) and for the interaction between the three factors studied (g). All acronyms used are described in the main text. Table S4: Variable importance in the PLS-R Projection (VIP). VIP > 1 are considered as the most important variables for the model. All acronyms used are described in the main text.
