# Supplementary material for: The Impact of Freeze-Drying Conditions on the Physico-Chemical Properties and Bioactive Compounds of a Freeze-Dried Orange Puree
Source: Foods. 2019 Dec 30;9(1):32. doi: 10.3390/foods9010032 (PMC7022254; doi:10.3390/foods9010032)
Supplement: Supplementary file 1 [file foods-09-00032-s001.pdf]

Supplementary Materials

Table S1. Values (mean ± standard deviation) of the different physicochemical properties evaluated. All acronyms used are described in the main text.

Sample	L *	C *	H *	ε (%)	S (N/mm)	F_f (N)	x_w (%)
S_30_P_5	83.1 ± 0.7	40.1 ± 0.9	81.4 ± 0.6	87.4 ± 0.5	15 ± 6	15 ± 4	3.18 ± 0.09
F_30_P_5	83.44 ± 1.58	43 ± 3	82.5 ± 0.9	86.6 ± 0.6	17 ± 6	20 ± 5	3.4 ± 0.2
S_30_P_{100}	80.45 ± 1.12	44 ± 2	80.5 ± 0.6	87.4 ± 0.8	11 ± 2	12.0 ± 1.6	3.96 ± 0.12
F_30_P_{100}	79.28 ± 1.18	44 ± 3	80.5 ± 0.7	87.6 ± 0.9	12 ± 7	14 ± 4	3.98 ± 0.13
S_40_P_5	84.9 ± 0.5	38.7 ± 1.8	81.9 ± 0.4	87.8 ±0 .4	16 ± 5	19 ± 6	2.9 ± 0.6
F_40_P_5	83.8 ± 0.8	42 ± 2	81.4 ± 0.5	87.2 ± 0.6	18 ± 5	17 ± 3	3.3 ± 0.7
S_40_P_{100}	80.7 ± 0.6	46.6 ± 1.6	80.5 ± 0.4	87.0 ± 0.6	17 ± 4	15.8 ± 1.2	4.2 ± 0.2
F_40_P_{100}	79.8 ± 0.9	51.9 ± 1.9	81.2 ± 0.4	86.7 ± 0.6	16 ± 4	17 ± 2	3.88 ± 0.08
S_50_P_5	83.51 ± 1.05	38 ± 2	81.5 ± 0.4	87.0 ± 0.7	26 ± 10	19 ± 4	2.2 ± 0.3
F_50_P_5	82.4 ± 0.7	47 ± 3	82.1 ± 0.5	87.3 ± 0.8	18 ± 8	16 ± 4	2.5 ± 0.4
S_50_P_{100}	80.9 ± 0.6	45.4 ± 1.9	81.3 ± 0.4	86.4 ± 0.4	18 ± 5	17.1 ± 1.9	2.93 ± 0.09
F_50_P_{100}	79.9 ± 0.8	51.2 ± 1.9	81.4 ± 0.4	86.6 ± 0.6	22 ± 7	17 ± 3	2.68 ± 0.13

Table S2. Percentage (mean ± standard deviation) of the bioactive compounds preserved in the FDP for each condition evaluated. All acronyms used are described in the main text.

Sample	TP*	VC*	BC*	DPPH*	FRAP*
S_30_P_5	90 ± 5	93 ± 6	65.5 ± 1.8	87.9 ± 0.5	99 ± 6
F_30_P_5	97.48 ± 0.05	96.7 ± 1.6	66 ± 3	86.81 ± 1.03	85 ± 11
S_30_P_{100}	91.6 ± 1.2	90.0 ± 0.3	27 ± 6	84.1 ± 0.8	93 ± 3
F_30_P_{100}	96 ± 3	89.8 ± 0.1	49.2 ± 1.9	90.5 ± 0.8	93 ± 3
S_40_P_5	87 ± 3	103.8 ± 1.5	65 ± 8	92 ± 5	94 ± 3
F_40_P_5	88 ± 4	104.7 ± 0.9	60 ± 2	93 ± 7	99 ± 4
S_40_P_{100}	89 ± 2	103.1 ± 0.9	39.8 ± 1.9	93 ± 2	97 ± 4
F_40_P_{100}	90.4 ± 1.8	104.09 ± 0.12	49 ± 10	94.3 ± 1.5	94 ± 2
S_50_P_5	93 ± 6	104.1 ± 0.7	50.73 ± 1.14	93 ± 4	96 ± 7
F_50_P_5	98.05 ± 0.17	103.8 ± 0.4	49± 3	93 ± 4	97.5 ± 0.8
S_50_P_{100}	92 ± 3	103.3 ± 0.2	40± 6	89 ± 2	99 ± 10
F_50_P_{100}	89.0 ± 1.9	102.4 ± 0.9	44± 10	86.5 ± 1.8	104 ± 6

* Bioactive compound preserved in the FDP in reference to the FOP (equation 7).

Table S3. Values (mean and p value according to Tukey'HSD test) of the different physicochemical properties and bioactive compounds evaluated for: each individual factor (a-c), the interaction between two different factors (d-f) and for the interaction between the three factors studied (g). All acronyms used are described in the main text.

a) **Summary (LS means)—Shelf temperature:**

	L*	C*	h*	F_f (N)	S (N/mm)	ε (%)	X_w (%)	TP (%)	DPPH (%)	FRAP (%)	BC (%)	VC (%)
30	81.568 b	42.730 b	81.238 a	15.414 a	13.706 b	87.256 a	3.618 a	93.855 a	87.345 b	92.436 b	52.064 a	92.305 b
40	82.317 a	44.776 a	81.243 a	16.984 a	16.644 b	87.178 a	3.571 a	88.660 b	93.307 a	95.976 ab	53.420 a	103.933 a
50	81.700 ab	45.306 a	81.570 a	17.487 a	20.859 a	86.843 a	2.575 b	93.021 a	90.510 ab	99.373 a	45.916 b	103.399 a
p-value	0.039	0.001	0.095	0.124	0.000	0.073	< 0.0001	0.003	0.001	0.034	0.008	< 0.0001

b) **Summary (LS means)—Pressure:**

	L*	C*	h*	F_f (N)	S (N/mm)	ε (%)	X_w (%)	TP (%)	DPPH (%)	FRAP (%)	BC (%)	VC (%)
P_5	83.534 a	41.418 b	81.792 a	17.761 a	18.444 a	87.227 a	2.900 b	92.364 a	91.127 a	95.173 a	59.313 a	100.977 a
P_{100}	80.189 b	47.123 a	80.908 b	15.496 b	15.695 a	86.958 a	3.609 a	91.326 a	89.648 a	96.683 a	41.620 b	98.781 b
p-value	< 0.0001	< 0.0001	< 0.0001	0.010	0.055	0.093	< 0.0001	0.385	0.195	0.459	< 0.0001	0.003

c) **Summary (LS means)—Freezing-rate:**

	L*	C*	h*	F_f (N)	S (N/mm)	ε (%)	X_w (%)	TP (%)	DPPH (%)	FRAP (%)	BC (%)	VC (%)
F	81.453 b	46.527 a	81.509 a	16.769 a	17.099 a	87.022 a	3.278 a	93.233 a	90.718 a	95.473 a	52.848 a	100.249 a
S	82.270 a	42.014 b	81.191 b	16.489 a	17.040 a	87.162 a	3.231 a	90.457 b	90.056 a	96.383 a	48.085 b	99.510 a
p-value	0.002	< 0.0001	0.025	0.741	0.967	0.376	0.638	0.027	0.556	0.655	0.018	0.275

Table S3 (cont.).

d) **Summary (LS means)—Shelf temperature*Pressure:**

	L*	C*	h*	F_f (N)	S (N/mm)	ε (%)	X_w (%)	TP (%)	DPPH (%)	FRAP (%)	BC (%)	VC (%)
30_P_5	83.268 ab	41.655 bc	81.962 a	17.800 a	16.338 ab	87.004 ab	3.268 b	93.731 ab	87.383 b	91.885 a	65.807 a	94.716 b
30_P_{100}	79.867 c	43.806 b	80.514 c	13.029 b	11.074 b	87.507 a	3.968 a	93.978 ab	87.307 b	92.986 a	38.321 c	89.893 c
40_P_5	84.388 a	40.266 c	81.635 a	17.777 a	16.908 ab	87.498 a	3.083 b	87.768 b	92.763 ab	96.590 a	62.499 a	104.254 a
40_P_{100}	80.246 c	49.286 a	80.850 bc	16.191 ab	16.379 ab	86.859 ab	4.060 a	89.551 ab	93.851 a	95.362 a	44.341 bc	103.613 a

50_P5	82.946 b	42.332 bc	81.780 a	17.706 a	22.086 a	87.178 ab	2.350 c	95.591 a	93.235 a	97.044 a	49.632 b	103.960 a
50_P100	80.453 c	48.279 a	81.359 ab	17.269 ab	19.632 a	86.508 b	2.800 bc	90.451 ab	87.785 ab	101.702 a	42.199 bc	102.838 a
p-value	0.038	< 0.0001	0.011	0.106	0.387	0.003	0.105	0.052	0.054	0.503	0.001	0.032

e) Summary (LS means)—Freezing-rate*Shelf temperature:

	L*	C*	h*	F_f (N)	S (N/mm)	ε (%)	X_w (%)	TP (%)	DPPH (%)	FRAP (%)	BC (%)	VC (%)
S_30	81.773 ab	41.798 b	80.956 b	13.893 a	12.866 b	87.376 a	3.568 a	90.811 ab	86.052 b	95.805 ab	46.470 b	91.371 b
F_30	81.363 b	43.663 b	81.520 ab	16.936 a	14.546 b	87.136 a	3.668 a	96.898 a	88.638 ab	89.066 b	57.658 a	93.239 b
S_40	82.795 a	42.655 b	81.224 ab	17.377 a	16.442 ab	87.397 a	3.563 a	88.022 b	92.842 a	95.344 ab	52.260 ab	103.479 a
F_40	81.839 ab	46.897 a	81.261 ab	16.592 a	16.845 ab	86.959 a	3.580 a	89.297 b	93.772 a	96.609 ab	54.580 ab	104.388 a
S_50	82.242 ab	41.588 b	81.392 ab	18.195 a	21.811 a	86.714 a	2.563 b	92.536 ab	91.275 ab	97.999 ab	45.526 b	103.680 a
F_50	81.157 b	49.023 a	81.747 a	16.780 a	19.907 ab	86.972 a	2.588 b	93.506 ab	89.745 ab	100.746 a	46.305 b	103.119 a
p-value	0.484	0.001	0.292	0.076	0.579	0.186	0.932	0.184	0.316	0.136	0.071	0.337

Table S3 (cont.).

f) Summary (LS means)—Freezing-rate*Pressure:

	L*	C*	h*	F_f (N)	S (N/mm)	ε (%)	X_w (%)	TP (%)	DPPH (%)	FRAP (%)	BC (%)	VC (%)
S_P_5	83.843 a	38.857 c	81.590 a	17.967 a	19.062 a	87.388 a	2.754 b	90.167 a	91.221 a	96.417 a	60.329 a	100.220 ab
F_P_5	83.225 a	43.978 b	81.994 a	17.555 a	17.826 a	87.066 a	3.047 b	94.560 a	91.033 a	93.929 a	58.297 a	101.733 a
S_P_{100}	80.697 b	45.170 b	80.791 b	15.010 a	15.018 a	86.937 a	3.708 a	90.746 a	88.892 a	96.349 a	35.842 c	98.799 b
F_P_{100}	79.680 c	49.077 a	81.025 b	15.983 a	16.372 a	86.978 a	3.510 a	91.907 a	90.404 a	97.018 a	47.399 b	98.764 b
p-value	0.425	0.302	0.541	0.416	0.360	0.253	0.018	0.182	0.451	0.439	0.001	0.254

g) Summary (LS means)—Freezing-rate*Shelf temperature*Pressure:

	L*	C*	h*	F_f (N)	S (N/mm)	ε (%)	X_w (%)	TP (%)	DPPH (%)	FRAP (%)	BC (%)	VC (%)
S_30_P_5	83.095 ab	40.070 def	81.403 bc	15.708 ab	15.214 ab	87.369 ab	3.175 bcd	89.979 a	87.959 ab	99.022 ab	65.524 ab	92.777 cd
F_30_P_5	83.442 ab	43.240 cde	82.520 a	19.892 a	17.462 ab	86.639 ab	3.360 bc	97.483 a	86.807 ab	84.749 b	66.091 a	96.655 bc
S_30_P_{100}	80.450 cd	43.527 cd	80.508 c	12.079 b	10.519 b	87.382 ab	3.960 ab	91.643 a	84.145 b	92.589 ab	27.416 f	89.964 d
F_30_P_{100}	79.283 d	44.085 cd	80.520 c	13.979 ab	11.629 b	87.632 ab	3.975 ab	96.312 a	90.469 ab	93.383 ab	49.225 bcde	89.822 d
S_40_P_5	84.928 a	38.696 ef	81.912 ab	18.911 ab	16.019 ab	87.788 a	2.886 cde	87.390 a	92.254 ab	93.684 ab	64.734 abc	103.816 a
F_40_P_5	83.848 ab	41.836 cdef	81.358 bc	16.644 ab	17.797 ab	87.209 ab	3.280 bcd	88.147 a	93.273 ab	99.496 ab	60.264 abcd	104.691 a
S_40_P_{100}	80.662 cd	46.613 bc	80.537 c	15.844 ab	16.865 ab	87.007 ab	4.240 a	88.655 a	93.431 ab	97.004 ab	39.785 ef	103.141 a
F_40_P_{100}	79.830 d	51.958 a	81.164 bc	16.539 ab	15.893 ab	86.710 ab	3.880 ab	90.447 a	94.271 a	93.721 ab	48.897 bcde	104.085 a
S_50_P_5	83.506 ab	37.806 f	81.456 bc	19.283 a	25.953 a	87.006 ab	2.200 e	93.133 a	93.449 ab	96.545 ab	50.728abcde	104.068 a
F_50_P_5	82.386 bc	46.858 bc	82.104 ab	16.129 ab	18.218 ab	87.349 ab	2.500 de	98.049 a	93.020 ab	97.543 ab	48.536 cde	103.853 a
S_50_P_{100}	80.978 cd	45.370 c	81.328 bc	17.108 ab	17.669 ab	86.422 b	2.925 cde	91.939 a	89.100 ab	99.454 ab	40.323 ef	103.291 a
F_50_P_{100}	79.928 d	51.188 ab	81.390 bc	17.430 ab	21.595 ab	86.594 ab	2.675 cde	88.962 a	86.471 ab	103.949 a	44.074 de	102.385 ab
p-value	0.268	0.133	0.004	0.316	0.080	0.305	0.468	0.284	0.184	0.062	0.272	0.420

Table S4. Variable importance in the PLS-R Projection (VIP). VIP > 1 are considered as the most important variables for the model. All acronyms used are described in the main text.

Variable	Component 1				Component 2			
	VIP	Standard Deviation	Lower Bound (95%)	Upper Bound (95%)	VIP	Standard Deviation	Lower Bound (95%)	Upper Bound (95%)
Pressure-P_{100}	1.808	0.110	1.588	2.027	1.444	0.089	1.267	1.620
Pressure-P_5	1.808	0.110	1.588	2.027	1.444	0.089	1.267	1.620
Shelf T 30	0.488	0.583	-0.673	1.649	1.218	0.243	0.734	1.702
Shelf T 50	0.378	0.459	-0.536	1.292	1.008	0.252	0.506	1.511
Shelf T 40	0.102	0.377	-0.649	0.853	0.193	0.364	-0.533	0.918
Freez-rate-S	0.191	0.354	-0.515	0.896	0.382	0.279	-0.174	0.938
Freez-rate-F	0.191	0.354	-0.515	0.896	0.382	0.279	-0.174	0.938
